# Applicability of Dmax Method on Heart Rate Variability to Estimate the Lactate Thresholds in Male Runners

**DOI:** 10.1155/2019/2075371

**Published:** 2019-09-19

**Authors:** Eduardo Marcel Fernandes Nascimento, Diego Antunes, Paulo Cesar do Nascimento Salvador, Fernando Klitzke Borszcz, Ricardo Dantas de Lucas

**Affiliations:** Physical Effort Laboratory, Sports Center, Federal University of Santa Catarina, Florianopolis, Brazil

## Abstract

**Introduction:**

The purpose of this study was to evaluate the application of the Dmax method on heart rate variability (HRV) to estimate the lactate thresholds (LT), during a maximal incremental running test (MIRT).

**Methods:**

Nineteen male runners performed two MIRTs, with the initial speed at 8 km·h^−1^ and increments of 1 km·h^−1^ every 3 minutes, until exhaustion. Measures of HRV and blood lactate concentrations were obtained, and lactate (LT_1_ and LT_2_) and HRV (HRVT_DMAX1_ and HRVT_DMAX2_) thresholds were identified. ANOVA with Scheffe's post hoc test, effect sizes (*d*), the bias ± 95% limits of agreement (LoA), standard error of the estimate (SEE), Pearson's (*r*), and intraclass correlation coefficient (ICC) were calculated to assess validity.

**Results:**

No significant differences were observed between HRVT_DMAX1_ and LT_1_ when expressed for speed (12.1 ± 1.4 km·h^−1^ and 11.2 ± 2.1 km·h^−1^; *p*=0.55; *d* = 0.45; *r* = 0.46; bias ± LoA = 0.8 ± 3.7 km·h^−1^; SEE = 1.2 km·h^−1^ (95% CI, 0.9–1.9)). Significant differences were observed between HRVT_DMAX2_ and LT_2_ when expressed for speed (12.0 ± 1.2 km·h^−1^ and 14.1 ± 2.5 km·h^−1^; *p*=0.00; *d* = 1.21; *r* = 0.48; bias ± LoA = −1.0 ± 1.8 km·h^−1^; SEE = 1.1 km·h^−1^ (95% CI, 0.8–1.6)), respectively. Reproducibility values were found for the LT_1_ (ICC = 0.90; bias ± LoA = −0.7 ± 2.0 km·h^−1^), LT_2_ (ICC = 0.97; bias ± LoA = −0.1 ± 1.1 km·h^−1^), HRVT_DMAX1_ (ICC = 0.48; bias ± LoA = −0.2 ± 3.4 km·h^−1^), and HRVT_DMAX2_ (ICC = 0.30; bias ± LoA = 0.3 ± 3.5 km·h^−1^).

**Conclusions:**

The Dmax method applied over a HRV dataset allowed the identification of LT_1_ that is close to aerobic threshold, during a MIRT.

## 1. Introduction

The autonomic cardiac drive can be investigated by the heart rate variability (HRV), which is characterized as a variation quantified in milliseconds between RR intervals [1]. A predominance of parasympathetic nervous system (PNS) activity is observed at rest and low effort intensities. In approximately 50–60% of the maximum oxygen uptake (VO_2MAX_), a significant vagal withdrawal occurs [2]. The aerobic threshold (AeT) has been related to that intensity [3–5], i.e., the exercise intensity which lactate concentrations [La] initiate to increase beyond resting values and are frequently called “lactate threshold” (LT) [6, 7]. On the other hand, in intensities above the AeT, there is a gradual and constant increase in activation in the sympathetic nervous system (SNS), and a marked increase in the physiological responses related to the anaerobic threshold (AnT) can be observed [3, 4]. That intensity is corresponding to maximal lactate steady state (MLSS), i.e., the highest constant exercise intensity output that can be maintained over time without continual [La] accumulation [3, 6].

Since AeT and AnT are good indexes of ideal training intensity and determinants of endurance performance [3, 4, 6], there is an obvious and growing interest in proposing different methods to estimate that intensities, mainly in relation to MSSL, considered gold standard endurance performance marker [3, 6, 8]. Among the different methods, the HRV thresholds (HRVTs) have been highlighted [5, 9–15]. The HRVT method is accessible and of low cost to use compared to traditional methods, such as [La] and gas exchange analysis, as well as a real noninvasive alternative to routine applications. The main methods for identifying the HRVTs are frequency domain [10, 12, 13], time domain [5, 9, 15], and nonlinear [10, 14] analysis. Success in identifying HRVTs and consequent estimation of AeT and AnT have been confirmed in different situations, such as in running [10–12], cycling [5, 9, 13–15], and ski mountaineering [16, 17], and also in high-level swimmers [18], trained boys [10], obese adolescents [19], and individuals with type 2 diabetes [20].

The Poincaré plot is a nonlinear HRV analysis method that uses time domain markers [21] and is an important research area, since it allows its use in nonstationary data, a characteristic inherent to HRV, especially during the increase of effort intensity [22]. The Poincaréplot analysis provides the calculation of the standard deviation of instantaneous (SD1) and continuous long-term RR intervals (SD2) [1]. The SD1 marker has been shown to correlate strongly with vagal tone (PNS), and previous studies have pointed to an abrupt point of change in their behavior in intensities related to AeT [2, 5, 9, 11]. SD2 marker has been shown to correlate with the PNS and SNS, and this variable shows a nonlinear pattern in intensities close to heavy and severe domains [2, 11]. The applicability and efficacy of the Poincaré plot in the estimation of the AeT and AnT have been confirmed in previous studies in running [11] and cycling [9, 15, 23].

However, in addition to the heart rate (HR) which presents theoretical support for a nonlinear pattern, especially in intensity close to AnT [24], some aspects need to be better elucidated when using HRV markers for the estimation of AeT and AnT. Firstly, it would be the validity of the method since the majority of studies used visual analysis for HRVT identification [9–12, 16], which is influenced by the subjective aspect and experience of the evaluator. In order to remedy this limitation, Cheng et al. [25] proposed the Dmax method to identify the lactate and ventilatory thresholds. Previous studies demonstrated greater reliability of the Dmax method than visual analysis or the use of fixed [La] [26]. The Dmax method presents an important advantage which a breakpoint can always be detected [25], although a maximal test is needed. Only one study of our knowledge used the Dmax method to identify HRVTs [23]. Their results surprisingly on the contrary, as reported by the authors, pointed to the visual analysis as better indicators of reliability in the SD1 and RMSSD (square root of the mean squared differences of successive RR intervals) markers than the Dmax method. This way, it is doubted if the Dmax method is better or not than the visual analysis for the identification of AeT or AnT, when using HRV dataset. Nevertheless, the results of the aforementioned study [23] were not compared with traditional methods to estimate AeT and AnT, as [La] or gaseous exchanges; therefore, greater inferences are limited. Secondly, no study to date has analyzed the possibility to identify the HRVTs by the Dmax method in the different situations and conditions compared to lactate and ventilatory thresholds. Finally, the reproducibility of the method must be verified in relation to different situations and conditions, since it has only been tested on the cycle [15, 23].

The identification of HRVTs and consequently the estimation of MLSS can be a framework very important to control and monitor training workloads, as well as to assess the improvement in performance during an endurance training program [21]. The applicability of HRV thresholds in a single-day test perhaps can be very attractive for research studies of sports science, trainers, and practitioners users. Therefore, the aim of the study is to investigate the application of the Dmax method originally proposed by Cheng et al. [25], on HRV dataset to estimate the AeT and AnT, during a maximal incremental running test (MIRT) in male runners. Firstly, the hypothesis is that the HRVT identified by SD1 marker (HRVT_DMAX1_) could be used to estimate the AeT, since this marker has been shown to correlate with the PNS [2, 9, 11, 15]. Secondly, the hypothesis is that the HRVT identified by SD2 marker (HRVT_DMAX2_) could be used to estimate AnT, since this marker has been shown to correlate with a significant PNS and mainly with the SNS [11]. The reproducibility of the HRVT_DMAX1_ and HRVT_DMAX2_, as well as AeT and AnT, will be verified.

## 2. Materials and Methods

### 2.1. Participants

Nineteen male recreational long-distance runners (30.4 ± 4.0 years; body mass of 74.3 ± 8.4 kg; height of 176 ± 6.3 cm; body fat of 13.8 ± 4.5%) volunteered to participate in this study. All participants were healthy, without cardiovascular or orthopedic problems, nonsmokers, and not taking any medication. The study protocol complied with the Declaration of Helsinki for human experimentation [27] and was approved by the Institutional Ethics Committee of the University of São Paulo.

### 2.2. Study Design

All the participants performed two MIRTs interspersed by a washout period of 3–7 days. The tests were performed at the same time of the day and in standard laboratory conditions (humidity of ≈50% and temperature of ≈22°C). Participants were instructed to avoid intense exercises, alcohol, and caffeine beverages 24 hours before each test and to consume a light meal 3 hours before the tests.

### 2.3. Maximal Incremental Running Test Protocol

Before MIRT, participants used a cardio belt for beat-to-beat heart rate (HR) measures (S810 Polar®, Kempele, Finland), during rest for 20 min (10 min supine + 10 min sitting) for baseline measures of HR, HRV, and [La]. The [La] was obtained from a 25 *μ*L blood, drawn from the tip of the forefinger, and blood samples were then stored in Eppendorf tubes containing 50 *μ*L of 1% NaF in a −30°C environment, according to the recommendations of the manufacturer (YSI 1500 Sport, Yellow Springs, OH, USA). Later, the samples were analyzed using enzyme electrode technology (YSI 1500 Sport, Yellow Springs, OH, USA). Then, the participants were directed on the treadmill (CEFISE TK35, Nova Odessa, Brazil) and warmed up for 3 min at 5 km·h^−1^ and 1% gradient. The test started at 8 km·h^−1^, with 1 km·h^−1^ increases every 3 min, until exhaustion, being a protocol adapted by Heck et al. [28]. The HR dataset was recorded continuously throughout the tests. Blood samples of 25 *μ*L were collected during the last 30 s of every stage, while the participant was running.

### 2.4. Determination of Aerobic and Anaerobic Thresholds

#### 2.4.1. Heart Rate Variability Thresholds

**Figure 1:**
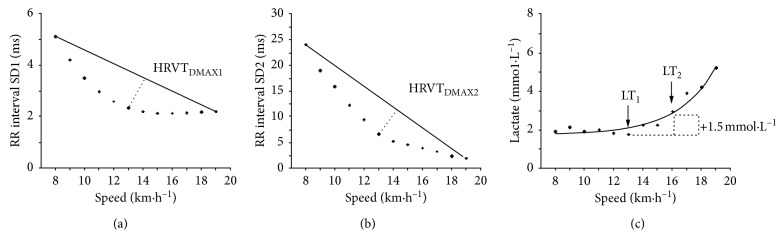
The HRVT_DMAX1_ (a) and HRVT_DMAX2_ (b) thresholds determined by the Dmax method and LT_1_ and LT_2_ (c) determined by lactate concentrations.

The Dmax method was used to analyze the behavior of SD1 and SD2 markers, to identify the HRVT_DMAX1_ and HRVT_DMAX2_, respectively (Figure 1). The Dmax method was determined according to Cheng et al. [25], thereby providing individualized lactate threshold values according to the following equation:

(1) $$\begin{array}{c} Y=a_{3}x^{3}+a_{2}x^{2}+a_{1}x+a_{0},\;\text{where }x\text{ is the workload }\left(\text{km}\cdot {\text{h}}^{-1}\right), \end{array}$$

where *Y* represents the predicted values of SD1 or SD2 at a given workload (km·h^−1^); *a*_*3*_, *a*_*2*_, *a*_*1*_, and *a*_*0*_ are the intercepts; and *x* is the speed. Briefly, the Dmax method reflects the longest perpendicular distance between SD1 and SD2 values predicted by a third-order polynomial function over actual (SD1 and SD2) values and values derived from a linear regression calculated with the first and last values of each curve, respectively. The SD1 marker was used because it has been shown to correlate strongly with vagal tone (PNS), and previous studies have pointed to an abrupt point of change in their behavior in intensities related to AeT [2, 5, 9, 11, 15]. SD2 marker has been shown to correlate with the PNS and SNS, and this variable shows a nonlinear pattern in intensities close to heavy and severe domains, being these related to AnT [2, 11]. Thereafter, raw RR intervals were recorded during the last 60 s of each stage of the exercise, and then the Dmax method was applied on the measured values.

#### 2.4.2. Lactate Thresholds

The first lactate threshold (LT_1_) (i.e., AeT) was determined as the lowest value of the ratio [La]/speed [29]. After, the second lactate threshold (LT_2_) (i.e. AnT) was determined as the running speed at 1.5 mmol·L^−1^ above LT_1_ (Figure 1) [29]. The LT_1_ and LT_2_ derived from the Dickhuth et al.'s [29] methods were used as criterium measures, because LT_1_ has a high correlation with MLSS [7], and LT_2_ (+1.5 mmol·L^−1^) was used because it showed a high concordance with MLSS in runners during the MIRT with stages of 3 min [30].

All the thresholds, HRVT_DMAX1_, HRVT_DMAX2_, LT_1_, and LT_2_, were expressed as absolute and relative values for speed (km·h^−1^), [La] (mmol·L^−1^), milliseconds beat-to-beat RR intervals (ms), and HR (bpm).

### 2.5. Statistical Procedures

Values were expressed as mean and standard deviation (±SD). After ensuring Gaussian data distribution (normality and homoscedasticity), a spreadsheet was used for the analysis of concurrent validity [31] and statistical standards were followed [32]. Cohen's [33] (*d*) effect sizes and ANOVA with Scheffe's post hoc test were used to compare the magnitude of the differences between the thresholds LT_1_ and LT_2_ with HRVT_DMAX1_ and HRVT_DMAX2_, respectively. Additionally, the standard error of the estimate (SEE), the bias ± 95% of limits of agreement [LoA] of the Bland and Altman analysis [34], and the Pearson product-moment correlation were used to evaluate the association between the different methods for identifying thresholds. For measures, reliability determination, the intraclass correlation coefficient (ICC), and the typical error of measurement (TEM) were performed using a Hopkins spreadsheet [31]. The *d* values were interpreted using the following scale: <0.20 (trivial), 0.2–0.6 (small), 0.6–1.2 (moderate), 1.2–2.0 (large), 2.0–4.0 (very large), and >4.0 (extremely large) [33]. Additionally, the ICC and the Pearson correlation coefficients (*r*) were interpreted as follows: <0.10 (trivial), 0.30 (small), 0.50 (moderate), 0.70 (large), 0.90 (very large), 0.99 (nearly perfect), and 1 (perfect) [31]. The data analysis was performed using the SPSS (19.0). The significance adopted was set at *p* < 0.05.

## 3. Results

### 3.1. Identification of HRVT_DMAX1_, HRVT_DMAX2_, LT_1_, and LT_2_

No significant differences were observed, neither for absolute nor for relative values, between HRVT_DMAX1_ and LT_1_ when expressed for speed (*p*=0.55; *d* = 0.45 and *p*=0.10; *d* = 0.76), lactate (*p*=0.24; *d* = 0.79 and *p*=0.13; *d* = 0.06), RR (*p*=0.54; *d* = 0.48 and *p*=0.50; *d* = 0.49), and HR (*p*=0.45; *d* = 0.44 and *p*=0.30; *d* = 0.51), respectively. In the same way, significant differences were not observed between HRVT_DMAX2_ and LT_2_ when expressed for lactate (*p*=0.13; *d* = 0.14 and *p*=0.99; *d* = 0.00) and RR (*p*=0.22; *d* = 0.68 and *p*=0.23; *d* = 0.68), but on the other hand, significant differences were observed when expressed for speed (*p*=0.00; *d* = 1.21 and *p*=0.00; *d* = 1.9) and HR (*p*=0.02; *d* = 1.15 and *p*=0.00; *d* = 1.24), respectively. Further, no significant differences were observed, neither for absolute nor for relative values, between HRVT_DMAX2_ and LT_1_ when expressed for speed (*p*=0.66; *d* = 0.41 and *p*=0.16; *d* = 0.75), RR (*p*=0.73; *d* = 0.35 and *p*=0.66; *d* = 0.70), and HR (*p*=0.61; *d* = 0.33 and *p*=0.61; *d* = 0.39).  Table 1 shows the results of all the methods.

Table 2 shows in detail the results of the Pearson correlation coefficient between the methods expressed as absolute and relative values for speed, lactate, RR, and HR.

**Figure 2:**
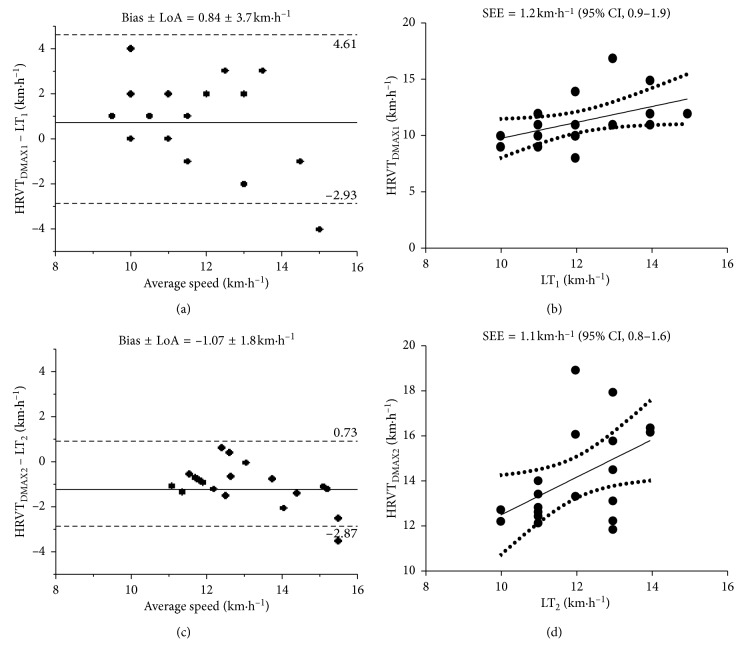
Bland and Altman plot for the agreement between HRVT_DMAX1_ and LT_1_ (a), and HRVT_DMAX2_ and LT_2_ (c), with bias (continuous line) and the 95% limits of agreement (discontinuous lines) for speed measures. The relationship between HRVT_DMAX1_ for speed and LT_1_ (b) and HRVT_DMAX2_ and LT_2_ (d) measures; solid and dashed lines represent the regression line and the 95% confidence intervals, respectively.

Figure 2 shows the magnitude of differences between HRVT_DMAX1_ and LT_1_, and HRVT_DMAX2_ and LT_2_. The Bland–Altman and regression analysis showed between HRVT_DMAX1_ and LT_1_ the bias ± LoA = 0.84 ± 3.7 km·h^−1^ and SEE = 1.2 km·h^−1^ (95% CI, 0.9–1.9), and between HRVT_DMAX2_ and LT_2_ the bias ± LoA = −1.07 ± 1.8 km·h^−1^ and SEE = 1.1 km·h^−1^ (95% CI, 0.8–1.6).

### 3.2. Reliability of the HRV and [La] Thresholds

In relation to baseline HRV values (423.0 ± 28 ms vs. 425.7 ± 25 ms; *p*=0.95; *d* = 0.09) and baseline lactate values (1.34 ± 0.4 mmol·L^−1^ vs. 1.25 ± 0.3 mmol·L^−1^. *p*=0.77; *d* = 0.41), no significant differences were observed between test and retest. With regard to values recorded at the exhaustion in MIRT, no significant differences were observed between test and retest for speed (16.4 ± 1.7 km·h^−1^ vs. 16.6 ± 1.7 km·h^−1^; *p*=0.96; *d* = 0.09), HR (192 ± 5 bpm vs. 191 ± 5 bpm; *p*=0.98; *d* = 0.12), and [La] (9.2 ± 1.8 mmol·L^−1^ vs. 8.2 ± 1.9 mmol·L^−1^; *p*=0.88; *d* = 0.52), respectively.

**Figure 3:**
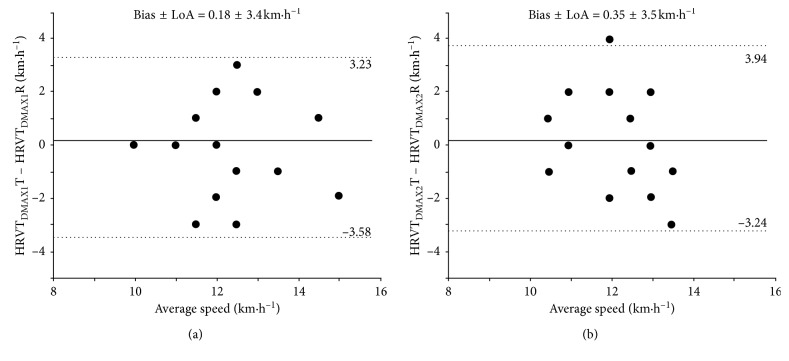
Bland and Altman plot for the reproducibility of HRVT_DMAX1_ (a) and HRVT_DMAX2_ (b) methods.

Significant differences were not observed between test and retest for all thresholds (HRVT_DMAX1_ = 12.1 ± 1.4 km·h^−1^ vs. 12.3 ± 1.5 km·h^−1^, HRVT_DMAX2_ = 12.0 ± 1.2 km·h^−1^ vs. 11.7 ± 1.4 km·h^−1^, LT_1_ = 11.2 ± 2.1 km·h^−1^ vs. 12.0 ± 1.5 km·h^−1^, and LT_2_ = 14.1 ± 2.1 km·h^−1^ vs. 14.2 ± 1.7 km·h^−1^). Large ICCs were found to HRVT_DMAX1_ when expressed in relation to RR and HR (ICC = 0.80-0.81; TEM = 5.1–4.9%, respectively). The Bland–Altman and regression analysis showed the bias ± LoA = 0.84 ± 3.7 km·h^−1^. In the same way, large ICCs were found to HRVT_DMAX2_ when expressed in relation to RR and HR (ICC = 0.80–0.82; TEM = 5.5–5.6%, respectively). Results further suggested a consistent reproducibility for LT_1_ and LT_2_, since the large to nearly perfect ICCs were showed for speed and HR measures (ICC = 0.90–0.97 and 0.80–0.94; TEM = 6.4–4.9% and 2.8–1.7%, respectively).  Table 3 shows all results of ICC and TEM for all the methods (Figure 3).

## 4. Discussion

The main findings of the present study were that the application of the Dmax method on HRV dataset (SD1 and SD2 markers by the Poincaré plot, being HRVT_DMAX1_ and HRVT_DMAX2_, respectively) enabled the estimation of the LT_1_ during a MIRT in male runners. However, the results refute one of the hypotheses of the study, which was that the HRVT_DMAX2_ method would estimate the AnT. The relative values found in HRVT_DMAX2_ and HRVT_DMAX1_ showed values with a greater approximation to the AeT. Consequently, it seems to suggest that when the Dmax method is applied to a HRV dataset extracted by the Poincaré plot, it is possible to identify a transition zone, with an approximation to the AeT, being an important intensity to improve cardiorespiratory and neuromuscular responses of runners. Furthermore, the results also suggested a consistent reproducibility for LT_1_ and LT_2_ (ICC =0.90 and 0.97, respectively), as well as, moderate to HRVT_DMAX1_ (ICC = 0.48) with low bias (−0.18 ± 3.4 km·h^−1^).

The results of the SD1 marker, which was used to identify the HRVT_DMAX1_, showed values of approximately ≈73.4% of the peak speed value, being these intensities related to the AeT [3, 4]. However, the values were slightly above the values reported in previous studies such as Garcia-Tabar et al. [9] analyzing a homogeneous group of professional male world-class road cyclists (≈36–52% W_PEAK_), Candido et al. [23] analyzing healthy individuals (≈50–60% W_PEAK_), Sales et al. [20] analyzing individuals with type 2 diabetes (≈64–66% VO_2PEAK_), and Tulppo et al. [2] investigating complete or not parasympathetic blockade. The HRVT_DMAX1_ has elicited signiﬁcant correlation when compared to LT_1_ (*r* = 0.46). The results of the present study in relation to HRVT_DMAX1_ are slightly below those found by Garcia-Tabar et al. [9], which used the same marker of PNS (SD1) to estimate the LT (*r* = 0.66–0.88), although different protocols and methodologies were applied. However, it is important to note that only a study of our knowledge by Nascimento et al. [11] used the same ergometer when analyzing HRV indices by the Poincaré plot and [La], in case the treadmill, and all other studies used a cycle ergometer. Consequently, greater comparisons are limits due mainly to the specificity and differences in the movement gesture as well as in the recruitment of motor units [35, 36].

It is important to note that the SD1 marker, which presents the prevalence of PNS activity, correlates with indices representing high-frequency bands (HF), such as in the Fourier Transform, when analyzing the behavior of HRV by frequency domain [1]. In addition, studies suggest that the respiratory pattern has a strong effect on the HF-HRV bands, both at rest and at exercise [37–39]. During exercise in heavy domain occurs an increase in respiratory frequency with a constant final volume, triggering a mechanical effect on the sinus node, concomitant with an increase in HF. This can be demonstrated in previous studies which identified changes in HF behavior below and above of ventilatory threshold [37].

The SD2 marker used in the present study to identify the HRVT_DMAX2_ showed significant differences in relation to LT_2_ when expressed for absolute and relative values of speed and HR, but not when expressed to lactate and RR, respectively. On the other hand, it is important to note that HRVT_DMAX2_ has elicited moderate coefficient values (*r* = 0.48) and signiﬁcant coefficient values (*r* = 0.71) when compared to LT_2_, with the values expressed for speed and HR, respectively. This variation in the correlations may be explained in part by the size and heterogeneous characteristic of the sample (body fat coefficient of variation (CV) is 33%). The values found in relation to the HRVT_DMAX2_ were approximately ≈72.9% of the peak speed value, being these intensities related to the AeT [3, 4]. Previous studies showed a breakpoint in SD2 to intensities of approximately ≈80%–90% VO_2MAX_ during maximal progressive cycling test [2] and intensities of approximately ≈86.1% of the peak speed value during MIRT [11]. However, as previously mentioned in relation to the possibility of identifying a single breakpoint by the Dmax method, these results suggest that the SD2 marker shows a nonlinearity behavior during a MIRT. Therefore, in addition to a breakpoint in intensity close to the AnT, as already demonstrated in previous studies [2, 11], a significant change point in SD2 also occurs at near intensities related to the AeT.

In the present study, the approximation of HRVT_DMAX2_ with the AeT, possibly, is due to the use of the Dmax method. This method allows the identification of only one breakpoint, although it is a nonsubjective method when compared to the visual method, for example. However, there are questions concerning the determination of the breakpoint by the Dmax method, in relation to the amount of values used in the model construction, being that the initial and final values can influence and compromise greater inferences when comparing the identification of the AeT or AnT [40].

Perhaps the use of different mathematical models [40] on HRV dataset, even with the possibility of submaximal tests [9], could allow greater accuracy in the estimation of AeT and AnT in different situations and populations involved. Moreover, both HRVT_DMAX1_ and HRVT_DMAX2_ are methods relatively simple to analyze and do not require a fixed number of RR intervals nor long recording periods [41], which facilitates the evaluation during incremental exercise. The usefulness of the HRVT_DMAX1_ and HRVT_DMAX2_ to estimate the AeT and determine aerobic capacity to prescribe exercise training intensities in sports performance and rehabilitation programs, from SD1 and SD2 values, is of great interest. The HRVTs may be objectively and noninvasively determined and are of lower cost than lactate or ventilatory threshold assessments, where blood lactate or gas analysis equipment is required as well as specialized professionals.

The LT_1_ and LT_2_ methods demonstrated a high level of reliability (ICC = 0.90 and 0.97; *p* < 0.001; TEM = 0.75 and 0.40 km·h^−1^; TEM = 6.4 and 2.8%, respectively). These results presented lower values than previous studies using similar markers for determination of LT_1_ and LT_2_ (TEM = 2.8 and 3.6%, respectively) [42]. Bland and Altman presented low values for difference between the test and retest as a function of their mean (bias ± LoA = −0.73 ± 2.0 km·h^−1^; bias ± LoA = −0.08 ± 1.1 km·h^−1^, respectively). Regarding the HRVT_DMAX1_, significant values were found in relation to RR and HR (ICC = 0.80 and 0.81; *p* < 0.001, respectively), but on the other hand, moderate values were found when expressed in relation to speed (ICC = 0.48), being below those found in a previous study (ICC = 0.73) using the same marker for identification of AeT [15], but through visual identification instead of the Dmax method. Regarding the TEM, results were slightly above the values found in the aforementioned study (TEM = 8.5%). Bland and Altman presented low values for difference between the test and retest as a function of their mean (bias ± LoA = −0.18 ± 3.4 km·h^−1^). Already to the HRVT_DMAX2_, significant values were found when expressed in relation to lactate (ICC = 0.60; *p* < 0.05) and RR and HR (ICC = 0.80 and 0.82; *p* < 0.001, respectively), with low values only when expressed in relation to speed (ICC = 0.30; *p* < 0.22), corroborating previous studies that used HRV markers by the Dmax method [23].

Due to its low cost, noninvasive nature, and high applicability, HRVTs is an important framework for researchers, trainers, and race practitioners. In the present study, its simplified and nonsubjective identification by the Dmax method suggests the possibility of planning a training program with a safe intensity in a metabolic transition zone, being slightly above the values found for AeT.

The present study certainly has some limitations that must be considered in the analysis of the results and their applicability. Aspects such as heterogeneous characteristic of the sample may try to explain in part the variation of correlation values between the different methods, since previous studies report that the greater the heterogeneity of a group, the greater the magnitudes of correlation [32]. Another fact may be just the recruitment of male runners and the need to perform a MIRT. In addition, MLSS was not used as a gold standard, but LT_2_ had a good approximation of MLSS in runners [30]. Therefore, it is suggested to carry out studies with different characteristics of samples, such as gender, age, and levels of training.

## 5. Conclusion

To the best of our knowledge, this is the first report on the application of the Dmax method on a HRV dataset to identify the lactate thresholds, during a MIRT. The results of this study showed that the Dmax method applied over a set of HRV during a MIRT in male recreational long-distance runners allowed the identification of HRVTs approaching the AeT. The HRVTs are of low cost, noninvasive nature, and high applicability. A limiting factor for the interpretation of the data is the recruitment of only males and trained, which do not allow the generalization of results to different populations. Thus, further studies are needed to confirm the reproducibility of HRVT as well as its use in different protocols, genders, age groups, and levels of training.

## 6. Disclosure

The level of evidence is Level II Study of Diagnostic Test.

## Figures and Tables

**Table 1:** Aerobic and anaerobic thresholds expressed as absolute and relative (mean ± SD) values for speed, blood lactate concentrations, milliseconds beat-to-beat RR intervals, and heart rate during maximal running test among trained male runners.

	Speed	Lactate				RR	HR	
	(km·h^−1^)	%^a^	(mmol·L^−1^)	%^a^	(ms)	%^b^	(bpm)	%^a^
HRVT_DMAX1_	12.1 ± 1.4^*∗∗*^	73.4 ± 7.3^*∗∗*^	2.2 ± 0.8	32.7 ± 9.4	379.4 ± 38	54.1 ± 5.2	159 ± 15^*∗*^	83.0 ± 7.7^*∗*^
(95% CI)	(11.4–12.7)	(69.9–77.0)	(1.8–2.6)	(28.2–37.2)	(360.9–398.0)	(51.6–56.6)	(151–167)	(79.3–86.7)
HRVT_DMAX2_	12.0 ± 1.2^*∗∗*^	72.9 ± 7.4^*∗∗*^	2.3 ± 1.2	33.4 ± 15.0	383.6 ± 41	54.6 ± 4.5	158 ± 17^*∗*^	82.2 ± 7.9^*∗∗*^
(95% CI)	(11.3–12.6)	(69.3–76.5)	(1.7–2.9)	(26.1–40.7)	(363.8–403.4)	(52.4–56.8)	(149–166)	(78.4–86.1)
LT_1_	11.2 ± 2.1^*∗∗*^	67.7 ± 7.6^*∗∗*^	1.7 ± 0.5^*∗∗*^	24.7 ± 8.5	397.9 ± 38^*∗∗*^	56.8 ± 5.6^*∗∗*^	151 ± 14^*∗∗*^	78.8 ± 6.3^*∗∗*^
(95% CI)	(10.2–12.3)	(64.1–71.4)	(1.4–2.0)	(20.6–28.8)	(379.3–416.5)	(54.0–59.5)	(144–158)	(75.8–81.9)
LT_2_	14.1 ± 2.1	85.2 ± 4.9	2.9 ± 0.5	33.5 ± 6.2	356.7 ± 37	51.0 ± 5.9	173 ± 9	90.0 ± 3.8
(95% CI)	(13.0–15.1)	(82.8–87.5)	(2.7–3.2)	(30.5–36.5)	(338.4–375.0)	(48.1–53.8)	(168–177)	(88.2–91.9)

^a^Percentage values are relative to maximal values during the MIRT. ^b^Percentage values are relative to 5 km·h^−1^ values (RR); CI = confidence interval. ^*∗∗*^*p* < 0.01 and ^*∗*^*p* < 0.05 in relation to LT_2_ method.

**Table 2:** Pearson correlation expressed as absolute and relative values for speed, blood lactate, RR interval, and heart rate during maximal running test among well-trained runners.

	Speed				Lactate				RR				HR			
	I	II	III	IV	I	II	III	IV	I	II	III	IV	I	II	III	IV
HRVT_DMAX1_ (I)	1	0.44	0.46^*∗*^	0.58^*∗∗*^	1	0.42	0.55^*∗*^	0.66^*∗∗*^	1	0.83^*∗∗*^	0.31	0.19	1	0.85^*∗∗*^	0.45	0.63^*∗∗*^
HRVT_DMAX2_ (II)	0.29	1	0.26	0.48^*∗*^	0.42	1	0.67^*∗∗*^	0.35	0.78^*∗∗*^	1	0.44	0.24	0.81^*∗∗*^	1	0.52^*∗*^	0.71^*∗∗*^
LT_1_ (III)	−0.18	−0.46^*∗*^	1	0.85^*∗∗*^	0.45^*∗*^	0.61^*∗∗*^	1	0.59^*∗∗*^	0.38	0.38	1	0.54^*∗*^	0.29	0.33	1	0.81^*∗∗*^
LT_2_ (IV)	−0.06	−0.27	0.41	1	0.29	−0.00	−0.08	1	0.36	0.28	0.66^*∗∗*^	1	0.57^*∗∗*^	0.55^*∗*^	0.54^*∗*^	1

HRVT_DMAX1_ = I; HRVT_DMAX2_ = II; LT_1_ = III, and LT_2_ = IV. Upper and lower triangles of each variable (speed, lactate, RR, and HR) refer to absolute and relative values, respectively. ^*∗*^*p* < 0.05. ^*∗∗*^*p* < 0.01.

**Table 3:** Reliability analysis for HRVT_DMAX1_, HRVT_DMAX2_, LT_1_, and LT_2_.

	Speed				Lactate				RR				HR			
	ICC		TEM^a^		ICC		TEM^a^		ICC		TEM^a^		ICC		TEM^a^	
	*M*	*p*	km·h^−1^	%	*M*	*p*	mmol·l^−1^	%	*M*	*p*	ms	%	*M*	*p*	bpm	%
HRVT_DMAX1_	0.48	0.08	1.18	9.6	0.42	0.12	0.67	29.6	0.80	0.00	19.4	5.1	0.81	0.00	8	4.9
HRVT_DMAX2_	0.30	0.22	1.21	10.1	0.60	0.29	0.80	36.4	0.80	0.00	21.8	5.6	0.82	0.00	8	5.5
LT_1_	0.90	0.00	0.75	6.4	0.73	0.00	0.33	19.2	0.75	0.00	21.6	5.5	0.80	0.00	7	4.9
LT_2_	0.97	0.00	0.40	2.8	0.71	0.00	0.32	10.6	0.49	0.08	24.2	6.9	0.94	0.00	2	1.7

^a^Multiply and divide these values by 1.4 to obtain the 95% confidence intervals (×/÷1.4). ICC, intraclass coefficient correlation; TEM, typical error of measurement.

## Data Availability

The data used to support the findings of this study are available from the corresponding author upon request.

## Abbreviations

HRV: Heart rate variability
VO_2MAX_: Maximum oxygen uptake
AeT: Aerobic threshold
PNS: Parasympathetic nervous system
SNS: Sympathetic nervous system
AnT: Anaerobic threshold
HRVT: Heart rate variability threshold
SD1: The standard deviation of instantaneous RR intervals
SD2: The continuous long-term RR intervals
HR: Heart rate
MIRT: Maximal incremental running test
LA: Lactate concentrations.
